# Aberration corrections for quasi-conformal transformation optics-based reflectors

**DOI:** 10.1038/s41598-025-30999-y

**Published:** 2025-12-09

**Authors:** Benjamin Cross, Gaeron Friedrichs, Dejan Filipovic

**Affiliations:** 1 Department of Electrical, Computer and Energy Engineering, University of Colorado, Boulder, 80303 USA; 2 Department of Electrical and Computer Engineering, University of Maryland, College Park, 20742 USA

**Keywords:** Engineering, Optics and photonics, Physics

## Abstract

Efficiency limitations of flat reflector antennas designed using quasi-conformal transformation optics are examined along with mechanisms to improve performance over wide bandwidths (> 2.5:1). Specifically, the aperture efficiency constraints are shown to be a result of phase aberrations induced by the approximate nature of the quasi-conformal transformation used to create an isotropic, non-magnetic structure. The aberrations are drastically reduced by introducing a simple curved surface into the domain prior to the transformation. This technique noticeably improves performance without increasing the size of the resulting device. Ultimately, this methodology allows for flat reflectors designed via quasi-conformal transformation optics to operate over wider bandwidths (up to 10:1) than with standard transformations.

## Introduction

For applications requiring high-directivity, reflector-based geometries find themselves in use across the spectrum^1^. In the context of modern-day systems, the relevance of reflectors at microwave and millimeter-wave (mmWave) frequencies is still unwavering^2,3^. With the proliferation of any technology, comes the inevitable efforts to reduce the size, weight, power, and cost (SWaP-C) of its practical implementation. For reflectors, one such method of great practical utility is the reflectarray^4^-^5^. Reflectarrays seek to enable the phase distribution required to produce highly-directive beams using low-profile reflective elements. They are often implemented using metallic structures on a single or multi-layer PCB^6–8^. While this drastically reduces the manufacturing burden, it reduces the bandwidth as well, since the elements typically only emulate the phase distribution accurately at a single frequency^9–11^. Attempts have been made to do this with dielectric elements as well, as this approach allows for lower cost of fabrication that can readily take advantage of modern manufacturing techniques such as 3D-printing^12–17^. To produce wider bandwidth, some approaches attempt to produce true time delay responses from the unit cells, mitigating the narrowband response often used to reproduce a specific phase shift^18–22^. In all these designs, the structures are placed in the region between the reflecting surface and the focal point.

A method left relatively unexplored is the utilization of transformation optics^23,24^ to solve this specific objective: a planar reflecting surface, with dielectric “grown” between the surface and the focal point, having wide bandwidth and faithfully emulating traditional reflector performance. Transformation optics, and quasi-conformal transformation optics have been explored in a number of antenna topics such as pattern control^25–28^, lens modification^29–33^, and cloaking^34,35^. While similar efforts have been discussed in literature^36,37^, the details are either insufficient, or some criteria has not been met (bandwidth, design details, etc.)^38–40^. An important consideration when attempting to synthesize dielectric devices is that, depending on the exact transformation, the resulting permittivity distribution may contain values less than unity. A common method of addressing this for wider-band, practical devices is through truncating the permittivity range, which—while potentially unavoidable—is known to impact performance^41,42^.

**Fig. 1:**
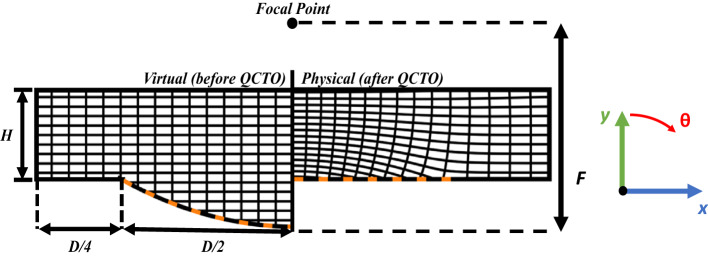
Baseline reflector geometry to be emulated (virtual space, pre-transformation - left) and lens reflector geometry (physical space, post-transformation - right). Reflecting surface denoted by orange dashed line.

This report discusses a quasi-conformal transformation optics-based approach to transform a parabolic reflector geometry into a planar geometry with dielectric placed between the reflecting surface and the focal point, as shown in Fig. 1. The proposed methodology demonstrates a novel device with improved performance over wide bandwidth. First, issues with the transformation process are identified as phase aberrations manifesting post-transformation. Next, two different techniques for mitigating the issues prior to the transformation process are proposed – one technique adapted from another application, and a novel approach. An analytically-defined cosine-shaped curve is placed on the top of the virtual space domain, resulting in a reduction of phase aberrations and wideband (10:1) operation as defined by the aperture efficiency of the structure.

## Transformation optics reflectors

### Quasi-conformal transformation optics

Performing a desired field transformation using standard transformation optics (TO) often results in material distributions requiring anisotropic permittivity and permeability tensors that are not realizable. However, quasi-conformal transformation optics (QCTO) allows for symmetric, isotropic, non-magnetic devices to be synthesized if certain constraints are met (Cauchy-Riemann conditions are approximately satisfied throughout the domain).

**Fig. 2:**
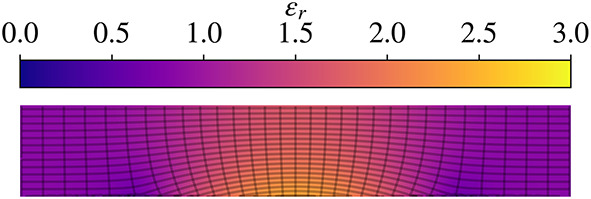
Representative permittivity distribution for the geometries under consideration.

In the context of practical devices and fabrication, the gradient-index of refraction (GRIN) profiles that result from QCTO enable wider bandwidths and lower losses than can be achieved with standard TO, and as such QCTO is explored here to design reflector antennas with a flat reflecting surface. Generally, QCTO mappings are determined in 2D for a TE polarization to preclude the need for magnetic materials. The resulting permittivity distributions (such as Fig. 2) are then revolved around their central axis to generate any number of different 3D devices^43–45^.

### QCTO solution process

The QCTO solution to flattening a prime-focus reflector is determined using COMSOL Multiphysics; the solution setup is illustrated in Fig. 1. The boundary conditions are imposed such that the parabolic surface in virtual space ($\:uv$) is “folded upward” into a plane in physical space ($\:xy$) that lies in-plane with the original reflector’s rim.

Performing QCTO requires solving Laplace’s equation for both $\:x(u,v)$ and $\:y(u,v)$. Neumann, or “slipping”, boundary conditions are applied to both $\:x(u,v)$ and $\:y(u,v)$ to allow the coordinate lines to slide, thus maintaining local orthogonality (and therefore isotropy). The distances between the rim of the reflector and the upper boundary (H) as well as between the edges of the reflector and the side boundaries determine the size of the solution domain. The distance between the edge of the reflector and the side boundaries is fixed to D/4 for all studies shown in this work, as it is far enough so as to not affect the coordinate mapping.

### Determining the permittivity profile

After obtaining the QCTO solution to the mapping between domains, the permittivity profile can be obtained by computing the Jacobian matrices of both the forward and reverse transformations and interpolating to the desired grid^46,47^. Practically, some computational noise is introduced, so a 50-point median filter is applied vertically to the profile. An example permittivity distribution generated through this process is shown in Fig. 2. The permittivity is highest in the center of the structure—where the spatial compression is highest—and tapers down towards the edge of the domain.

## Full-wave performance of QCTO reflectors

### Simulation setup

The generated 3D lens models are electrically-large (D > 10λ), so simulations are performed with a quarter-model (two symmetry planes) in CST Studio Suite. Critically, the permittivity distributions used in these full-wave simulations do include values below 1, and thus are not realizable without narrow-band metamaterial implementations. For practical fabrication, these regions can be replaced with permittivity values of 1 or re-scaled to ensure the optics are preserved^48^.

To excite the QCTO reflector, a unidirectional, Huygens source was used^49^. This classical, numerical source provides a constant excitation over frequency, and eliminates aperture blockage along with any other phenomenon that may mask the lensing behavior (multiple reflections, variation vs. frequency, etc.). As a result, this numerical feed is expected to perform better with lower F/D geometries due to the reduced spillover losses.

### F/2-Height lens

**Fig. 3:**
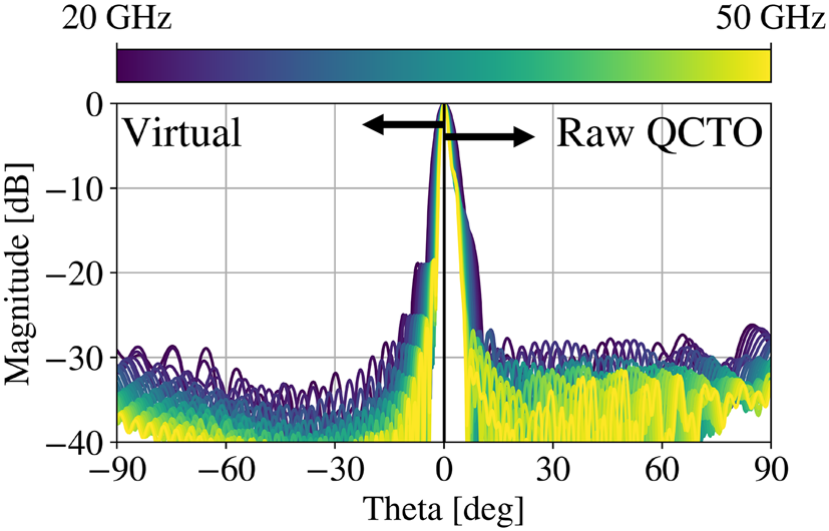
Comparison of magnitude and phase impact of the scattered co-pol. fields on the surface of the lens.

A compressed reflector geometry is used to benchmark these studies. The resulting values of the parameters in Fig. 1, are described in Table 1. The permittivity profile of this lens is illustrated in Fig. 2. As a performance benchmark, the radiation patterns for the QCTO reflector (Fig. 3, right) are compared against those of the equivalent paraboloidal system (Fig. 3, left). The presence of shoulders and bulging in the patterns of the QCTO system indicate that there is an issue with the amplitude/phase on the lens aperture.

**Table 1:** Reflector parameter values for the annotated variables in Fig. 1.

Parameter	F/D	D	F	H
Value	0.5	200 mm	100 mm	50 mm

Due to the broadened beams, the QCTO reflector demonstrates a significant reduction in aperture efficiency relative to the geometry it is intended to emulate: the virtual achieves a stable aperture efficiency of about 45% over the band versus 20–30% aperture efficiency with the QCTO approach. To faithfully replicate the performance of the virtual, the amplitude/phase on the aperture must be studied.

### Diagnosing aperture efficiency reduction

**Fig. 4:**
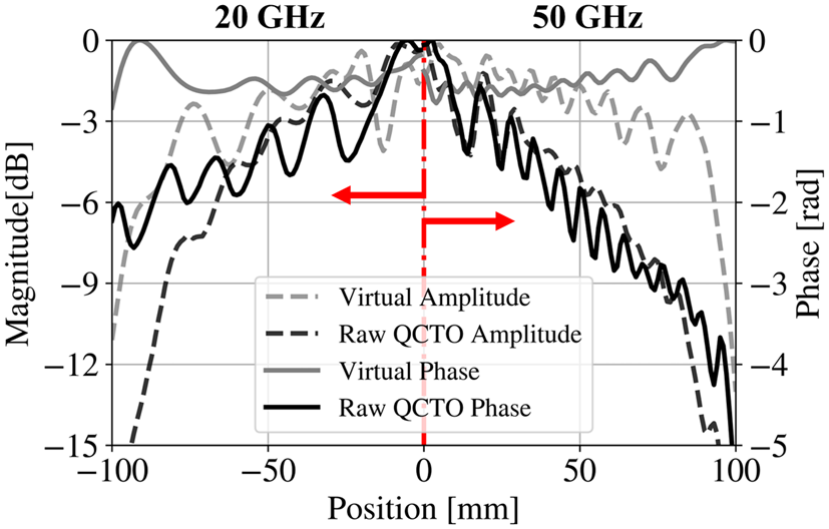
H-plane radiation patterns of the virtual parabolic reflector and the QCTO lens as designed from 20–50 GHz.

The near-fields of the QCTO reflector are studied as a proxy to diagnose its far field deficiencies. The fields scattered by the compressed reflector are directly compared to those of the parabolic reflector, by first extracting the amplitude and phase of the numerical solutions’ fields above the lens. Comparisons of the amplitude and phase distributions at 20 and 50 GHz are shown in Fig. 4.

This figure suggests that the QCTO reflector possesses amplitude and phase distributions with more taper and variation than the baseline geometry (paraboloid). The quantitative impact can be observed by synthesizing the far-fields, which can be performed using the near-field to far-field (NF-FF) transform. The fields from the Raw QCTO result are then separated into their amplitude and phase components, and an idealized amplitude and phase is substituted in, respectively, prior to the transformation, to yield two additional results.

**Fig. 5:**
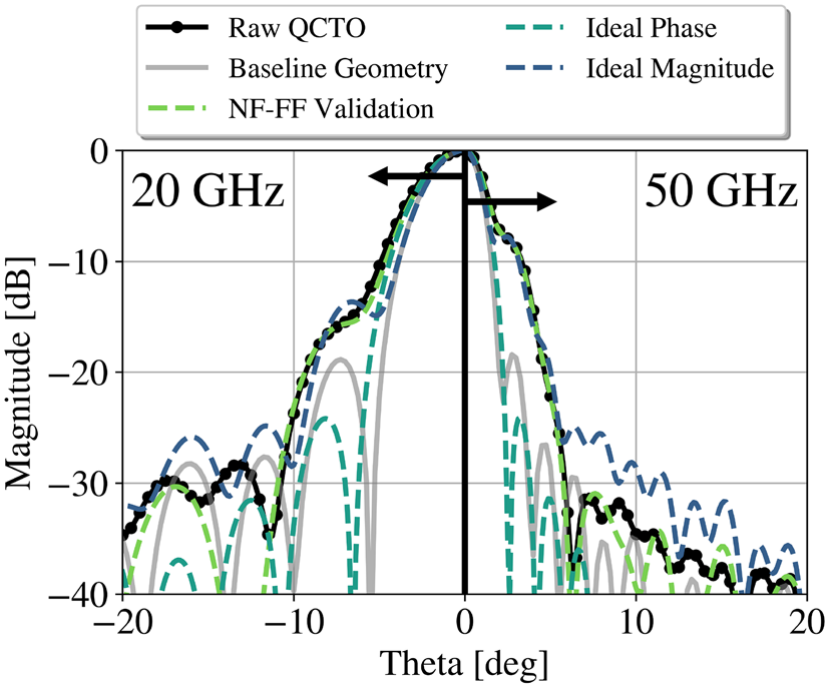
Relative effects of amplitude and phase distribution on the far-fields of the QCTO reflector at 20 GHz and 50 GHz.

Far-fields computed in this manner are demonstrated in Fig. 5. From the component-wise diagnosis, the phase distribution is shown to have a more significant impact, which must be remedied – ideally prior to the transformation.

## Correcting phase aberrations

### Methods for correcting aberrations

The default, compressed reflector geometry generated through QCTO does not accurately emulate the true time-delay operation of parabolic reflectors. In general, correcting for phase aberrations of an arbitrary radiating structure is accomplished by shaping a reflecting or refracting surface such that the resulting phase front is flat. Certain works consider QCTO to create corrective lenses tailored to the phase front of a given antenna^50^. This concept is applied here to include an additional lensing surface in the virtual domain while leaving the size of the physical device unchanged. Ultimately, this additional lensing surface applies a correction to the permittivity profile throughout the solution domain.

### Spline-based lensing surface

Based on the findings in^47^, the scattered phase distribution generated by the lens is used to determine the phase front shape needed. One basic approach can be performed by sampling the physical phase front in the air above the lens (“Phase Front”). This yields a physical curve that can be included in the QCTO solution domain.

Once the surface is obtained, the inverse coordinate mapping must be used to map the phase front back to virtual space while considering the spatial dilation/compression that occurs as a result of the slipping boundaries applied during the QCTO procedure. A 7th-order polynomial-fit, based on fields sampled at 50 GHz is used.

**Fig. 6:**
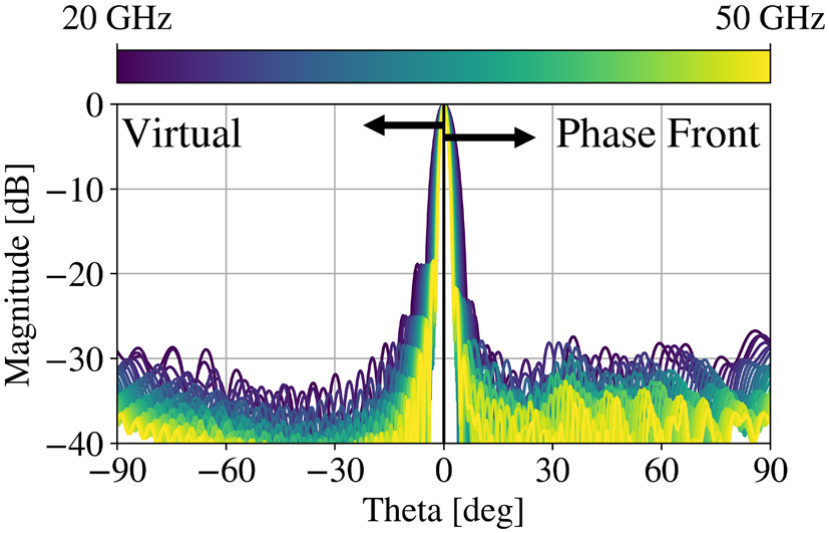
H-plane radiation patterns of the virtual parabolic reflector and the phase-front corrected QCTO lens as designed from 20–50 GHz.

The resulting lens is simulated in CST using the same setup as the original compressed reflector, the results for which are shown in Fig. 6. The phase front correction significantly improves the aperture efficiency performance. Though a novel application of the technique in^47^, this approach does require burdensome simulation time/resources to obtain the fields that are used to generate a correcting surface that must be added back in to the original domain. Thus, a straightforward, analytically-defined approach is desired to allow a designer to make the correction without multiple solutions.

### Simple geometric surface

**Fig. 7:**
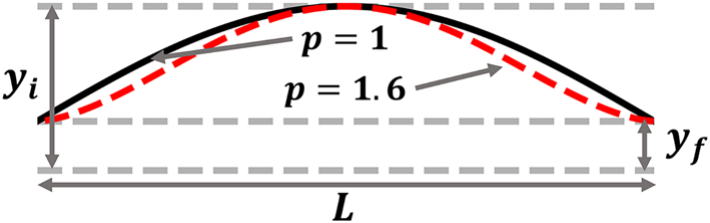
Simple geometrically-parameterized surface using a cosine-p shape. This curve sits atop the solution domain (virtual space) to provide an analytically-defined phase-correcting surface.

In contrast to the surface designed using the phase front of the QCTO reflector, the proposed solution uses a cosine-p curve as the upper boundary of the QCTO solution domain. This modified boundary is parameterized in terms of its height at the center of the domain ($\:{y}_{i}$), height at the edge of the domain ($\:{y}_{f}$), and the power to which the cosine is raised (p); the last of which allows for additional control over the slope. The top of the new transformation domain is illustrated in Fig. 7 with the phase-correcting surface included.

1 $$\:y={y}_{f}+\left({y}_{i}-{y}_{f}\right){\mathrm{cos}}^{\mathrm{p}}\left(\frac{\pi\:x}{2L}\right)$$

**Fig. 8:**
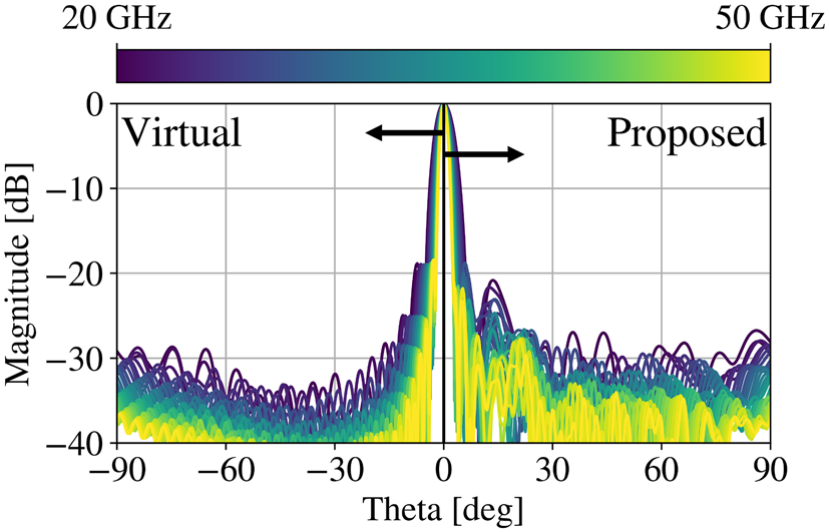
H-plane radiation patterns of the virtual parabolic reflector and the lens resulting from the proposed technique as designed from 20–50 GHz.

In this case, the curve shown in Fig. 7 would be place on top of the top boundary shown in Fig. 1 (left). The resulting transformation would still yield a device in the form factor of Fig. 1 (right). First, the edge height, $\:{y}_{f}$, is set to be 0 while the center height and exponent are allowed to vary. The center height is swept ($\:F/100$ to $\:F/6.67$) with a fixed exponent of 1 for the first study. Then, the center height is fixed to $\:F/50$ while the exponent is varied from 0.5 to 20. As the height of the cosine surface is increased, the average aperture efficiency increases until it reaches a maximum at $\:{y}_{i}=F/25$. Increasing the additional surface height past this point reduces aperture efficiency as a result of the diverging phase front being “over-corrected”, thus leading to fields in the center of the lens being out-of-phase with those on the edges. Taking a correcting surface of this height and changing p yields a similar trend; *p*=1 ultimately provides the best aperture efficiency over a wide bandwidth. The radiation patterns of the resulting lens are shown in Fig. 8.

**Fig. 9:**
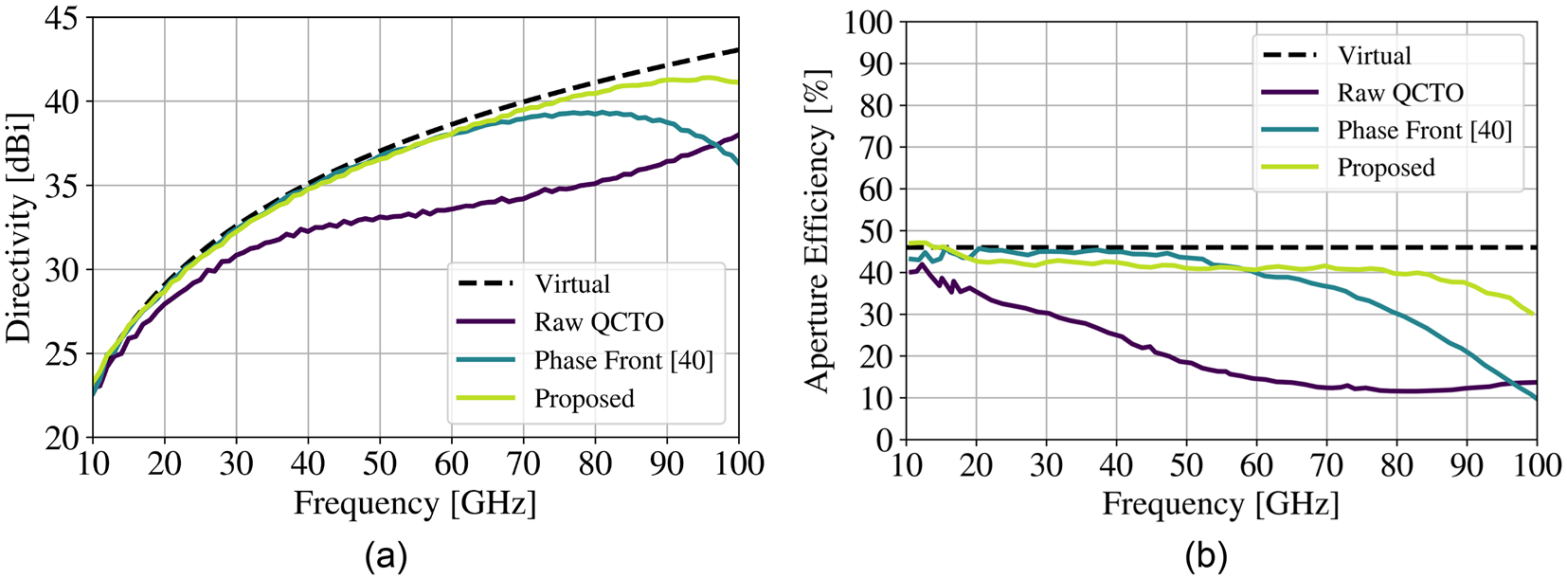
Reference directivity of the baseline structure (dashed) as well as the resulting lens results for different aberration mitigation techniques (**a**), as well as the corresponding aperture efficiency curves (**b**).

The resulting patterns demonstrate stable aperture efficiency over the band. This technique has the benefit of simplicity. It is worth noting, however, that the sidelobe behavior changes - distinct sidelobes emerge further away from the main beam, albeit with > 20 dB suppression with respect to the main lobe at all frequencies. The proposed method also sees benefits in the bandwidth of the aberration mitigation beyond the originally studied 2.5:1 relative bandwidth. An additional study conducted over 10:1 bandwidth (Fig. 9) demonstrates the enhanced relative bandwidth preservation.

These results demonstrate a distinct advantage over other implementations. The proposed process enables low sidelobe levels, high gain and aperture efficiency, with wide bandwidth all within the context of a 3D structure. Other techniques are compared in Table 2.

**Table 2:** Comparison of similar works.

References	Bandwidth	Dimensionality	Gain	Sidelobe level
^36,37^	3.5:1	2D	N/A	N/A
^38^	2.3:1	3D	29.55 dBi	< -15 dB
^39^	1.3:1	2D	N/A	< − 7 dB
^40^	2:1	2D	N/A	< -9 dB
Proposed	10:1	3D	41 dBi	< − 20 dB

**Fig. 10:**
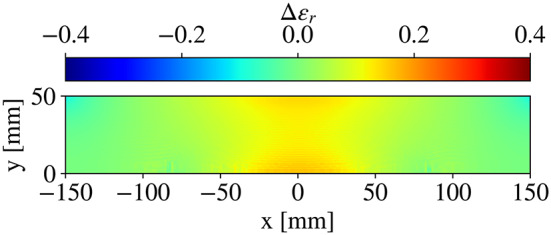
Difference in permittivity profiles from the original and the cosine-treated (proposed) lens.

A careful inspection of the final lens as compared to the raw QCTO lens is shown in Fig. 10. An increase in core permittivity is observed to help correct phase aberrations, but the increase of $\in _{r}$ is less than 0.3 – this is ultimately a relatively small change contained within the original form factor. The proposed approach demonstrates a much wider bandwidth of operation, without requiring an iterative, full-wave-intensive approach.

### Feed integration impacts

Thus far, the studies shown have utilized an idealized feed (Huygens source) to mitigate the impact of shadowing, scattering, standing waves, and other impacts observed when a feed is placed in front of a reflector. The net effect on performance in the far field is influenced by the relative sizes of the reflector, feed, as well as the specific antenna performance of the feed (ridged waveguide horn, corrugated horn, log-periodic antenna, phased-array, etc.). Other considerations of this problem in its entirety are inherently their own limiting factors in terms of performance. The work demonstrated in this report alleviates the performance burden from the technique itself, and ultimately moves the performance limitation back to being dominated by the traditional reflector considerations, thus implying a faithful recreation of conventional reflector performance.

**Fig. 11:**
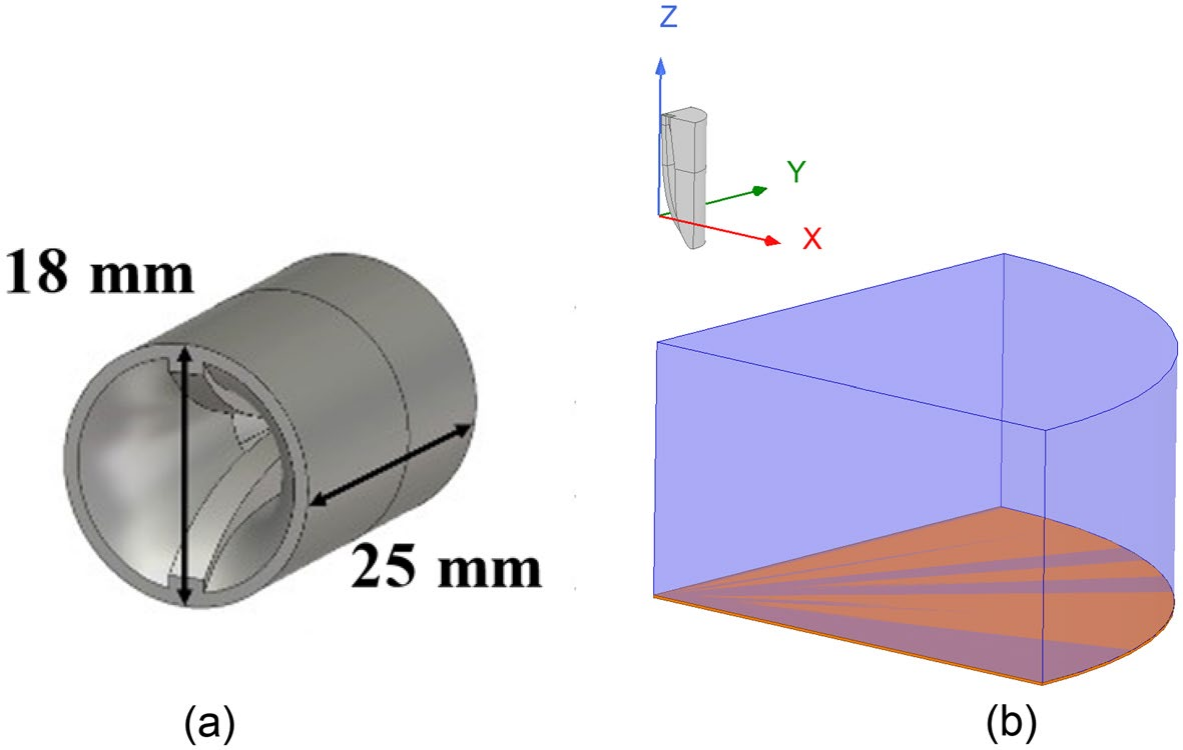
A realistic feed integration utilizing (**a**) a dual-ridge horn covering 20-50 GHz and (**b**) a full-wave lens model with prescribed permittivity gradient according to Fig. 10.

However, inclusion of a realistic feed is a natural extension of the proposed technique. A dual-ridge, conical horn was selected as the reflector feed, with the model shown in Fig. 11a. The feed was utilized to illuminate the lens developed in Fig. 10. An important distinction is that the feed was simply placed above the lens, as shown in Fig. 11b, replacing the Huygens source. The lens itself was not regenerated, and no further optimization was conducted to ensure seamless integration with a realistic feed.

**Fig. 12:**
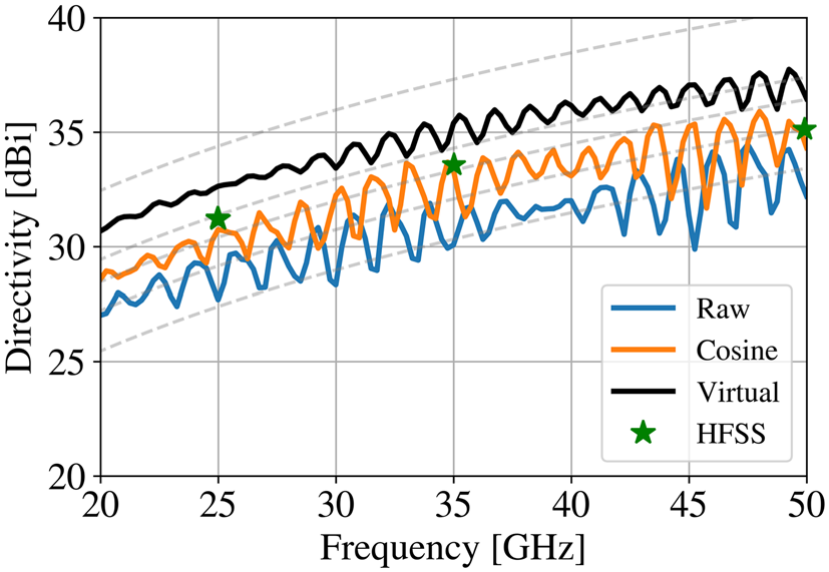
Directivity results for the virtual (baseline) geometry as well as the raw lens and treated (proposed) lens. HFSS validation shown.

The far field directivity results are shown in Fig. 12. The feed was evaluated when integrated with the baseline reflector (*virtual*, before transformation), the standard QCTO lens (*Raw*, no treatment applied), and the proposed technique (*Cosine*, correcting surface). The results of the proposed technique were further validated utilizing FEM technique, in addition to the time domain technique used to evaluate all the previous results. It is clear that the trend holds between the results in Fig. 9 and the results presented here. Degradation is overall performance is seen due to the integration of an unoptimized feed, and a standing wave can be observed in the far field.

In order to mitigate the impacts seen in Fig. 12, the procedure outlined in this manuscript can be reused. However, the calculation of the permittivity profile should be done with a virtual reflector and feed design that is as realistic as possible. This, however, is outside the scope of this particular report.

## Conclusion

This work investigated methods for addressing aperture efficiency degradation that occurs during the QCTO design process for flattened reflector antennas. Phase-correcting surfaces are added to the virtual domain to address this issue, following both a method in existing literature and a simplified parameterized method proposed here. The proposed method demonstrates significantly improved bandwidth characteristics compared to both the raw QCTO-derived device and the device corrected using a surface derived directly from the phase front. This design procedure can be used to enable realizations of practical flattened reflector antennas for wideband (10:1) applications, and only requires the generation of a single solution.

## Data Availability

Data is provided within the manuscript.
